# Managing Breakthrough Fungal Infections in Hematologic Patients: Determinants and Practical Management from a Latin American Perspective on Behalf of INFOCUS LATAM–ISHAM Working Group

**DOI:** 10.3390/microorganisms14040904

**Published:** 2026-04-16

**Authors:** Larissa Simão Gandolpho, Daniel Aguilar-Zapata, Pablo Andrés Moncada-Vallejo, Fernando Riera, Mariana Guaraná, Giovanni Luis Breda, Ricardo Rabagliati, Marcio Nucci, Arnaldo Lopes Colombo

**Affiliations:** 1 Division of Infectious Diseases, Escola Paulista de Medicina da Universidade Federal de São Paulo, São Paulo 04023-062, Brazil; lari.gandolpho@hotmail.com; 2 Hospital Nove de Julho, São Paulo 04023-062, Brazil; 3 Infectious Diseases and Hospital Epidemiology, Mexico City 14080, Mexico; daniel_aguilar@hotmail.com; 4 Department of Internal Medicine, Fundacion Valle del Lili, Cali 760032, Colombia; drpmoncada@gmail.com; 5 Division of Infectious Diseases, Sanatorio Allende, Córdoba X5000, Argentina; friera@hotmail.com; 6 Research Group of Immunology and Mycology, Córdoba X5000, Argentina; 7 Facultad de Ciencias Médicas, Universidad Nacional de Córdoba, I Catedra de Infectología, Córdoba X5000, Argentina; 8 Hospital Universitário Gaffrée e Guinle, Universidade Federal do Estado do Rio de Janeiro, Rio de Janeiro 20270-001, Brazil; mguarana@me.com; 9 Hospital de Clínicas, Universidade Federal do Paraná, Curitiba 80060-900, Brazil; giovanni.breda@hc.ufpr.br; 10 Department of Infectious Diseases, Faculty of Medicine, Pontificia Universidad Católica de Chile, Santiago 8331150, Chile; rabagli@gmail.com; 11 Red de Salud UC CHRISTUS, Santiago 8320000, Chile; 12 Division of Hematology, Internal Medicine Department, Universidade Federal do Rio de Janeiro, Rio de Janeiro 21941-913, Brazil; mnucci@hucff.ufrj.br; 13 Grupo Oncoclínicas, Rio de Janeiro 22250-905, Brazil; 14 Antimicrobial Resistance Institute of São Paulo (ARIES), São Paulo 04023-062, Brazil

**Keywords:** breakthrough fungal infections, antifungal prophylaxis failure, invasive mold infections, *Fusarium* spp., *Aspergillus* spp., mucormycosis, rare yeasts (*Saprochaete*; *Trichosporon*; *Rhodotorula*), drug resistance, therapeutic drug monitoring, acute myeloid leukemia, hematologic malignancies, hematopoietic stem cell transplantation

## Abstract

Breakthrough invasive fungal infections (bIFIs) are a challenging serious complication in high-risk hematologic patients and allogeneic hematopoietic stem cell transplantation recipients that may negatively impact their outcome. Despite advances in antifungal prophylaxis, diagnostics, and supportive care, bIFI occurrence reflects a complex interaction between host immunosuppression, emergence of resistant pathogens and pharmacological variables, including subtherapeutic drug exposure. *Candida* spp. have shifted towards non-*albicans* yeasts, whereas breakthrough mold infections more frequently involve non-fumigatus *Aspergillus*, Mucorales, *Fusarium* spp., and *Scedosporium*/*Lomentospora* spp. Early clinical recognition, rapid therapy escalation, aggressive diagnostic investigation, a switch to liposomal amphotericin B-based regimens in patients on azole prophylaxis, and therapeutic drug monitoring are essential to improve outcomes. Reducing the growing global burden of bIFIs will also require improved access to high-quality diagnostics and strengthened educational and stewardship efforts that prioritize antifungal resistance as an urgent health concern.

## 1. Introduction

Invasive fungal infections (IFIs) remain a major complication among patients with hematologic malignancies and hematopoietic stem cell transplantation (HSCT) recipients, with epidemiology evolving significantly over recent decades [1,2]. Although invasive candidemia (IC) and invasive aspergillosis (IA) remain the most frequent IFIs, an increasing proportion of infections are now caused by non-*Candida* yeasts and non-*Aspergillus* molds, with substantial geographic variability [3,4,5,6,7].

Despite major advances in non-culture-based diagnostics, imaging techniques, antifungal therapies, and preventive strategies that have substantially reduced IFI-associated morbidity and mortality, a subset of high-risk patients continues to develop breakthrough invasive fungal infections (bIFIs) while receiving antifungal treatment [1,2,8,9,10]. These events have been associated with diagnostic uncertainty, antifungal resistance, and poorer outcomes, representing a complex growing challenge in hematology practice [11,12]. Our INFOCUS LATAM panel of specialists prepared a narrative review for supporting good clinical practices in the clinical management of bIFIs based on an extensive search for PubMed, Scopus, and SciELO articles published in English between 1 January 1995 and 31 October 2025. Search terms included combinations of keywords and controlled vocabulary related to breakthrough invasive fungal infections, candidemia, aspergillosis, hematologic malignancies, hematopoietic stem cell transplantation, antifungal prophylaxis, antifungal resistance, diagnostic biomarkers, therapeutic drug monitoring, and emerging antifungal agents.

## 2. Strategies for Early Initiation of Antifungal Therapy in High-Risk Hematologic Patients

Because the risk of IFI is heterogeneous among hematologic populations, three main strategies (prophylactic, empiric, and preemptive [or diagnostic-driven]) have been adopted based on individual risk stratification to identify patients most likely to benefit from early antifungal intervention [13,14,15,16,17]. In acute leukemia, IFI risk is primarily driven by the intensity and duration of neutropenia, severity of mucositis, and relevant comorbidities [13,15,18]. Among allogeneic HSCT recipients, determinants vary according to the transplant phase [14]. Pre-engraftment risk includes stem cell source, conditioning intensity, T-cell depletion, and environmental exposures, whereas post-engraftment risk is largely associated with graft failure, graft-versus-host disease (GVHD), cytomegalovirus (CMV) reactivation, and cumulative immunosuppression [13,14,15].

In scenarios with a lower incidence of mold infections, or when mold-active azole prophylaxis is contraindicated due to toxicity or drug–drug interactions (DDIs), a diagnostic-driven approach may be reasonable [6,15,18]. This strategy typically combines fluconazole prophylaxis with active surveillance, including serial serum galactomannan antigen (GM) testing and early chest computed tomography (CT) in patients with persistent or unexplained fever, rather than initiating empirical antifungal therapy solely on the basis of fever [15,19,20]. Preemptive approaches have been shown to be safe and significantly reduce antifungal exposure without increasing mortality or IFI incidence [17,21]. In contrast, patients at higher risk for mold-related IFI generally benefit from primary prophylaxis with mold-active azoles, particularly posaconazole or voriconazole, with isavuconazole emerging as a reasonable alternative based on evidence from randomized clinical trials (RCTs) and real-world cohorts [15,18,22]. Finally, empiric antifungal therapy should be considered with caution and reserved mainly for clinically unstable patients while further diagnostic investigations are ongoing, or in centres lacking adequate diagnostic resources [20,23,24].

## 3. Breakthrough Invasive Fungal Infections

bIFIs pose significant diagnostic and therapeutic challenges, with reported incidence varying widely across studies [11]. RCTs evaluating antifungal prophylaxis in patients with acute myeloid leukemia/myelodysplastic syndrome (AML/MDS) and GVHD have reported bIFI rates ranging from 2 to 5%, whereas observational cohorts show broader variability, with incidences between 3% and 30% [11,25,26]. This variability reflects heterogeneity in patient risk profiles, antifungal classes used, pharmacological factors such as drug exposure and tissue penetration, and standards of supportive care, including environmental protection and catheter-care practices [12,27,28]. In addition, comparisons across studies are further limited by the lack of standardized definitions in earlier reports, resulting in limited knowledge of the true burden of bIFIs and optimal management strategies [11].

To improve comparability across studies, the Mycoses Study Group Education and Research Consortium (MSG-ERC) and the European Confederation of Medical Mycology (ECMM) proposed consensus definitions for breakthrough IFI to be applied alongside the EORTC/MSGERC criteria for proven, probable, and possible IFI [29]. According to these definitions, bIFI is defined as a new proven, probable, or possible IFI occurring during exposure to an antifungal agent, regardless of treatment intent (prophylactic, empiric, preemptive, or targeted). Importantly, the onset of bIFI is determined by the first attributable clinical, radiological, or mycological evidence occurring after a minimum period of antifungal exposure sufficient to achieve steady-state drug concentrations [29,30].

Geography substantially impacts the epidemiology of fungal infections. In Latin America, *Aspergillus* spp. remain the leading cause of invasive mold infections; however, fusariosis appears disproportionately frequent and, in some centres, more common than mucormycosis [6,8]. Candidemia also remains a significant complication, frequently involving non-*albicans Candida* spp. such as *C. tropicalis* and *C. parapsilosis* [6,7,8]. Additionally, rare yeasts are increasingly recognized in the region, including invasive infections due to *Trichosporon* spp., typically associated with poor outcomes, and fungemia caused by *Rhodotorula* spp. [5,9].

Several factors contribute to the complexity of bIFI management. Exposure to antifungal therapy can reduce the diagnostic performance of cultures, serologic tests, and PCR-based assays in this setting, particularly fungal biomarkers such as GM, and it may also select for intrinsically resistant or acquired-resistant pathogens, thereby narrowing therapeutic options [10,11,12,13,14]. These challenges are compounded by major disparities in diagnostic capacity and antifungal availability across regions. Although some tertiary centres have access to fungal biomarkers, MALDI-TOF MS, molecular diagnostics, antifungal susceptibility testing, and therapeutic drug monitoring (TDM), access to these tools, as well as to key antifungal agents such as liposomal amphotericin B (L-AmB) and echinocandin, remains inconsistent in many settings [10,13,15,16].

In parallel, uncontrolled or relapsing underlying hematologic disease limits immune recovery and increases susceptibility to infection [27,30,31,32,33,34]. Given the profound immunosuppression of these patients, a broad differential diagnosis must also be considered when evaluating sepsis, pulmonary infiltrates, or neurological symptoms, including viruses, bacteria, *Mycobacterium* spp., *Nocardia* spp., and fungi [35,36,37]. Collectively, these factors highlight the need for an aggressive, comprehensive diagnostic workup to accurately identify bIFI etiology and guide timely, effective antifungal therapy.

## 4. Breakthrough Yeast Infections

*Candida* spp. and related genera within the *Saccharomycotina* subphylum, particularly *Candida albicans*, are major components of the human mycobiome, colonizing mucosal surfaces, the gastrointestinal (GI) tract, and the skin [38]. Disruption of epithelial barriers combined with immune dysfunction related to chemotherapy, mucositis, or GVHD facilitates microbial translocation and invasive disease, contributing to mortality rates of 20–50% among hematologic patients [39,40].

In the pre-fluconazole era, *C. albicans* was the predominant cause of fungemia. In the early 1990s, after the first RCT demonstrated that fluconazole prophylaxis significantly reduced candidemia incidence, from 16% to 2.8%, it became broadly adopted in high-risk populations [41]. Therefore, subsequent multicentre cohort studies, including those from the EORTC and EBMT, confirmed these findings, reporting cumulative incidences below 1% in selected patient groups [3,42]. As a result, bIFIs caused by azole-susceptible *Candida* spp. are now predominantly observed in patients with refractory/relapsed malignancies, delayed engraftment after HSCT, or severe acute/chronic GVHD with prolonged immunosuppression [42,43,44,45,46]. Over the past two decades, selective pressure from widespread antifungal use has driven a marked shift in yeast epidemiology. Currently, more than half of yeast-related bIFIs are caused by triazole-resistant pathogens, including non-*albicans Candida* spp. and other emerging rare yeasts [47,48,49].

### 4.1. Breakthrough Infection by Candida *spp.* and Related Genera

The species distribution of breakthrough candidemia has shifted markedly toward non-*albicans Candida* spp., a pattern consistently reported across contemporary cohorts, without a single dominant species [2,3,49]. The most frequently isolated include *Nakaseomyces glabratus* (syn. *C. glabrata*), members of the *C. parapsilosis* complex, *C. tropicalis*, and *Pichia kudriavzevii* (syn. *C. krusei*), several of which exhibit intrinsic or acquired antifungal resistance. For instance, in a large cohort of 144 episodes of IC in cancer patients, *C. glabrata* was the predominant pathogen, with 10% demonstrating echinocandin resistance and up to 20% showing azole resistance, underscoring the growing therapeutic challenge posed by this species. Less frequent yeasts, including *C. kefyr*, *C. guilliermondii*, *C. dubliniensis*, and the emerging multidrug-resistant (MDR) *C. auris*, are increasingly recognized and represent important targets for institutional surveillance programmes [3,45,47,48,49,50,51,52].

### 4.2. Breakthrough Infection by Non-Candida (and Non-Cryptococcus) Yeasts

Beyond *Candida* spp., bIFIs caused by non-*Candida* and non-*Cryptococcus* yeasts are increasingly reported in hematologic and HSCT populations. The most frequently implicated genera include *Trichosporon* spp., *Rhodotorula* spp., *Magnusiomyces* (syn. *Saprochaete*/*Geotrichum* spp.), and *Saccharomyces* spp., with fungemia rates ranging from 0.7% to 4.1% in retrospective cohorts [4,43,53,54,55,56,57,58,59]. Data from Europe, Latin and North America consistently identify *Trichosporon asahii* (~20–50%) and *Rhodotorula mucilaginosa* (~10–50%) as leading pathogens, followed by *Magnusiomyces* (syn. *Saprochaete*/*Geotrichum* spp.) and *Saccharomyces* spp., highlighting both wide geographic variability and global convergence in species distribution [53,57,60].

These organisms are of particular concern in these populations due to their virulence, frequent resistance to echinocandins, and limited susceptibility to azoles or amphotericin B [55,61,62,63]. Moreover, misidentification or delayed diagnosis remains common once accurate identification often requires specialized methods such as MALDI-TOF mass spectrometry or molecular assays [64,65,66,67,68]. Thus, in suspected breakthrough fungemia during echinocandin exposure, rare yeasts should be systematically considered, and prompt therapeutic adjustment guided by species identification and antifungal susceptibility testing (AST) is essential. Many of these pathogens also form catheter-associated biofilms, making early central venous catheter removal a critical component of management [69].

### 4.3. Potential Impact of Low Gastrointestinal Echinocandin Concentrations

Echinocandins are recommended as first-line therapy for IC owing to their potent activity against most *Candida* spp., favourable safety profile, and minimal drug–drug interactions (DDIs) [70,71,72]. However, substantial interindividual variability in pharmacokinetics has been observed, particularly in critically ill, obese, and oncologic patients, leading to inconsistent systemic exposure [71,72,73,74]. In a prospective pharmacokinetic study of micafungin in adults with and without cancer, patients with malignancies exhibited faster drug clearance and an increased risk of underexposure, especially when treating *Candida* spp. isolates with higher MICs [75].

An additional limitation is their poor penetration into the GI tract, resulting in subtherapeutic luminal concentrations despite adequate plasma levels [76,77,78]. In the presence of mucosal damage (e.g., mucositis or GVHD), insufficient gut exposure may facilitate yeast translocation and may partially explain bIFIs occurring during echinocandin use [44,45,79,80]. Supporting this concern, cohorts of hematologic patients developing bIFIs during echinocandin exposure have increasingly reported echinocandin-resistant *Nakaseomyces glabratus* (syn. *C. glabrata*), *Candida parapsilosis*, and *Trichosporon asahii*, highlighting the need to integrate pharmacological considerations, pathogen-specific susceptibility, and host factors when managing suspected breakthrough yeast infections [76,77,78,80].

### 4.4. Management of Breakthrough Fungemia in Hematologic Malignancy or HSCT

Breakthrough fungemia requires early suspicion and a structured management approach, integrating clinical assessment, rapid diagnostics, empirical escalation guided by prior antifungal exposure, and prompt transition to targeted therapy [27]. Clinical suspicion should arise in high-risk patients with fever and hemodynamic instability, or rapid clinical deterioration despite broad-spectrum antibiotics, particularly in centres with known prevalence of resistant *Candida* spp. or rare yeasts [23,70].

#### 4.4.1. Initial Clinical Assessment and Diagnostic Workup

A prompt and comprehensive diagnostic workup should include the collection of 2–3 sets of aerobic blood cultures (approximately 60 mL in adults), followed by AST of all recovered isolates [81,82].

To optimize the diagnostic approach for suspected bIFI, clinicians should consider utilizing blood culture bottles specifically designed for fungal detection as a complementary tool alongside standard aerobic cultures in selected cases. These specialized bottles have demonstrated broader and often superior performance, achieving 100% detection for *Candida* spp. and significantly shorter times to detection (TTDs) for emerging pathogens like *Nakaseomyces glabratus* and *Candidozyma haemuli*, as well as filamentous fungi such as *Aspergillus terreus* and *Neocosmospora solani* [81,82].

Additional investigations, such as targeted imaging and tissue sampling with culture and AST from skin or organ lesions, should be pursued whenever feasible to distinguish isolated fungemia from deep-seated or disseminated infection [81,82].

Rapid species-level identification (with MALDI-TOF or molecular assays) is critical, as intrinsic resistance patterns and susceptibility profiles vary widely and directly influence therapeutic choices and prognosis [66,70].

#### 4.4.2. Empirical Management Before Pathogen Identification

**Figure 1:**
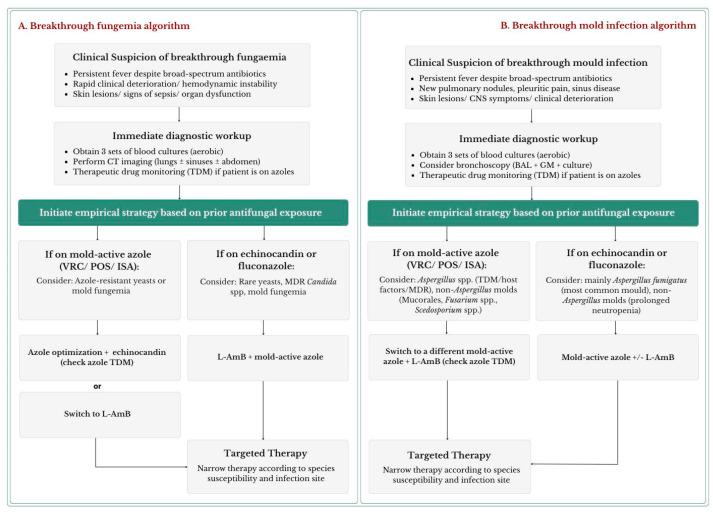
Initial management algorithms for suspected breakthrough invasive infections in hematologic patients and HSCT recipients. These complementary algorithms provide frameworks for the early recognition and management of suspected breakthrough (**A**) fungemia and (**B**) mold infection, highlighting rapid diagnostic workup and empirical escalation guided by prior antifungal exposure until proper species identification and antifungal susceptibility testing become available to estabilish targeted and definitive treatment. Abbreviations: BAL = Bronchoalveolar lavage, GM = Galactomannan, CT = Computed tomography, HSCT = Hematopoietic stem cell transplant, GVHD = Graft-versus-host disease, TDM = Therapeutic drug monitoring, ISA = Isavuconazole, VRC = Voriconazole, POS = Posaconazole, L-AmB = Liposomal amphotericin B.

While microbiological results are pending, early escalation of antifungal therapy should be guided by prior antifungal exposure to anticipate the most likely pathogens and minimize delays in effective treatment [66,70]. As illustrated in Figure 1A, this initial management algorithm combines rapid diagnostic evaluation with empirical escalation until species identification and AST allow targeted and definitive therapy.

In patients receiving mold-active azoles (voriconazole, posaconazole, or isavuconazole), breakthrough fungemia most often reflects azole-resistant *Candida* spp. or infections occurring in the context of subtherapeutic azole exposure, often related to inadequate therapeutic drug monitoring (TDM) (e.g., rare yeasts such as *Trichosporon* spp. and mold fungemia, particularly *Fusarium* spp.) [15]. Empirical strategies include azole optimization guided by TDM combined with an echinocandin or switching to liposomal amphotericin B (L-AmB). Although L-AmB exhibits variable activity against *Trichosporon* spp., monotherapy may be reasonable in settings with low local prevalence of these pathogens [81,83].

By contrast, breakthrough fungemia during echinocandin or fluconazole prophylaxis is more frequently associated with MDR *Candida* spp., rare yeasts, and mold fungemia [66,70]. Accordingly, escalation to L-AmB, often combined with a mold-active triazole, is generally recommended, particularly in patients with severe disease or profound immunosuppression [66,70].

#### 4.4.3. Targeted Therapy and Source Control

Once species identification and AST results are available, therapy should be narrowed to the most active and least toxic agent, and source control optimized, including catheter removal and drainage of focal infections [19,51,66]. In cases of persistent fungemia or involvement of sanctuary sites such as the central nervous system or vitreous, reassessment is required, given the limited penetration of echinocandins. In these scenarios, fluconazole, voriconazole, or L-AmB should be preferred when supported by susceptibility data and clinical status [66,70].

## 5. Breakthrough Mold Infection

In RCTs, breakthrough invasive mold infections have been reported in approximately 1–3.7% of patients, whereas real-world cohorts describe incidences of up to 11%, reflecting marked heterogeneity in host immune status, environmental exposure, and prophylactic strategies [25,84,85]. Compared with primary invasive mold infection, bIFIs are more often caused by molds with variable or reduced susceptibility to antifungal agents and are consistently associated with higher mortality rates [1,12,86].

Supporting these findings, in a systematic review including 1076 bIFIs occurring during voriconazole and posaconazole prophylaxis, non-*fumigatus Aspergillus* spp. predominated, followed by Mucorales, *Fusarium* spp., and *Scedosporium*/*Lomentospora* spp. [11]. However, in routine clinical practice, many bIFIs lack mycological confirmation and are classified as possible IFIs, as illustrated by a prospective Italian cohort of 260 patients with AML, in which bIFIs were reported in 17.2% of cases, the majority without mycological documentation [87]. In this context, early recognition relies heavily on clinical suspicion, imaging, and integration of host- and treatment-related factors.

Certain clinical features may help narrow the differential diagnosis. Compared with IA, mucormycosis is more strongly associated with prior voriconazole exposure, prolonged neutropenia, extended corticosteroid use, sinus involvement, multiorgan dissemination, and hemoptysis [11,88,89]. Fusariosis, on the other hand, more often presents with disseminated skin lesions, frequent fungemia, and pulmonary nodules, whereas serum galactomannan positivity is less consistent than in IA [90,91,92,93,94,95]. Although none of these findings are pathognomonic, their recognition can inform early empirical decisions while definitive diagnostics are pursued.

### 5.1. Determinants of Mold Breakthrough Infection

Breakthrough mold IFIs result from a complex interaction between host-related, fungal, and pharmacologic factors [12,96]. Understanding these determinants is essential to interpret prophylaxis failure and to guide rational rescue strategies.

#### 5.1.1. Host-Related Factors

A growing proportion of mold IFIs now occur late after HSCT, particularly in patients with chronic GVHD, underscoring the role of immune dysfunction [27,96]. Profound or prolonged neutropenia, lymphodepletion, and impaired T-cell-mediated immunity compromize both innate and adaptive antifungal responses, reducing the host’s ability to contain molds and to prevent angioinvasion [27,96]. Therefore, bIFIs may occur despite mold-active prophylaxis and adequate plasma antifungal concentrations [97]. Also, environmental exposure also plays a role and high airborne conidial burden, particularly in centres without HEPA filtration or during hospital construction, may overwhelm prophylactic strategies in patients with delayed immune recovery [27,28]. Finally, conditions related to disruption of the GI barrier can also impair drug absorption, and further facilitate fungal invasion [12,27,98].

#### 5.1.2. Fungal-Related Factors

Intrinsic resistance to prophylactic agents directly shapes the etiologic pattern of bIFI. Voriconazole prophylaxis is more frequently followed by Mucorales and non-fumigatus Aspergillus spp., whereas posaconazole failures more often involve *Aspergillus* spp. and *Fusarium* spp. [11,25].

Biofilm formation on airway or necrotic tissue can further reduce antifungal penetration and impair prophylaxis efficacy [27,99]. Although still relatively uncommon in Latin America, acquired azole resistance in *Aspergillus fumigatus* has been increasingly documented worldwide, both during prolonged therapy and in association with environmental exposure to agricultural azoles [100,101,102].

#### 5.1.3. Pharmacological Factors

Azoles are extensively metabolized through cytochrome P450 enzymes, especially CYP3A4. Concomitant use of enzyme inducers may substantially reduce antifungal exposure, whereas azole-mediated CYP3A4 inhibition can increase toxicity from hematologic therapies [103,104]. Optimising management of DDIs is therefore essential in patients receiving chemotherapy, targeted agents (venetoclax, BTK inhibitors), or immunosuppressants to ensure adequate levels of these drugs [104,105,106]. Moreover, TDM is particularly important for triazoles with unpredictable pharmacokinetics, such as voriconazole and posaconazole. For voriconazole, target trough concentrations between 1.0 and 5.5 mg/L are generally recommended to balance efficacy and toxicity, with higher targets (e.g., ≥2.0 mg/L) often considered in severe infections. For posaconazole, trough levels above 0.7 mg/L are recommended for prophylaxis and above 1.0 mg/L for treatment. Although isavuconazole exhibits more predictable pharmacokinetics, TDM may be considered in selected cases, such as treatment failure or infections caused by pathogens with elevated minimum inhibitory concentrations [83,104].

In outpatient settings or where only posaconazole oral suspension is available, adherence and absorption remain critical issues, as missed doses or incorrect administration may lead to subtherapeutic levels and bIFIs [105,107].

### 5.2. Management of Breakthrough Mold Infections

Early recognition of breakthrough mold infection is crucial, as presentations are often nonspecific. Suspicion should arise in patients on mold-active prophylaxis who develop persistent fever despite broad-spectrum antibiotics, new pulmonary or sinus lesions on computed tomography, acute skin nodules, or central nervous system manifestations [23,37,108]. These patients require immediate clinical reassessment and an aggressive diagnostic approach, including high-resolution imaging and invasive procedures such as bronchoscopy with bronchoalveolar lavage (BAL), nasal endoscopy, or tissue biopsy when feasible [30,88,109].

#### Empirical Management Before Pathogen Identification

Once breakthrough IMI is suspected, determinants of the likely etiology should be rapidly assessed by integrating host factors, prior antifungal exposure, and pharmacological considerations while diagnostic investigations are initiated [25,109]. Empirical antifungal therapy should not be delayed and is best guided by prior prophylaxis, as summarized in Figure 1B, which provides an initial exposure-driven management algorithm and emphasizes high-resolution imaging, bronchoscopy, serial biomarkers, and empiric therapy tailored to the likelihood of resistant or intrinsically less susceptible molds.

In patients receiving mold-active azoles, *Aspergillus fumigatus* remains predominant during early high-risk phases, particularly when subtherapeutic azole exposure occurs due to inadequate TDM. By contrast, prolonged immunosuppression favours intrinsically less susceptible molds, including non-fumigatus *Aspergillus*, Mucorales, and *Fusarium* spp. [25,97]. In this setting, empirical therapy should include L-AmB, with or without a switch to an alternative mold-active azole, and prompt assessment of azole plasma concentrations.

In patients receiving echinocandins or fluconazole, *A. fumigatus* is the most likely pathogen, but non-*Aspergillus* molds should also be considered in cases of prolonged neutropenia or profound immunosuppression. Empirical therapy should include a mold-active triazole, with early escalation to L-AmB in patients with severe disease or high suspicion of resistant pathogens. Breakthrough IMI during amphotericin B exposure should raise concern for non-*fumigatus Aspergillus* spp. or intrinsically resistant molds such as Mucorales, *Fusarium* spp., or *Scedosporium* spp. warranting empirical therapy with a mold-active triazole while diagnostic workup continues.

Regardless of the determinant factors involved, early empirical escalation to a broad-spectrum regimen (most commonly LAmB) should be considered until the probable pathogen is defined [25]. Supporting this approach, a multicentre Spanish cohort reported antifungal modification in over 90% of bIFI cases, most frequently involving a class switch to L-AmB [38].

## 6. Diagnostic Considerations in Breakthrough IFI

Serum GM remains the main fungal biomarker for early diagnosis of IA and may also support the diagnosis of fusariosis, although sensitivity is variable (0–83%) [91,93,110,111]. Nonetheless, bronchoscopy with BAL plays a central role in patients with pulmonary infiltrates, enabling simultaneous evaluation for fungal, bacterial, viral, and mycobacterial pathogens, which frequently coexist in hematologic patients [31,36,112,113]. Also, meta-analyses of GM in BAL report high diagnostic accuracy of BAL galactomannan, particularly when serum biomarkers are negative (pooled sensitivity and specificity of 0.79 and 0.92, respectively), being a key diagnostic tool for IA when serum biomarkers are negative or inconclusive [114]. In patients with cutaneous lesions, tissue biopsy is strongly recommended, as direct microscopy may reveal characteristic features such as adventitious sporulation in *Fusarium* spp. or *Lomentospora prolificans* infections [86,115,116].

Species-level identification and AST are essential to guide targeted therapy and safe intravenous-to-oral transition [32,113]. Despite this, AST data are reported in a minority of bIFI cases, even though resistance to the prophylactic agent is common [15]. This underscores the importance of access to reference laboratories, molecular diagnostics, and robust AST in routine practice [117,118].

In addition to conventional microbiological methods and fungal biomarkers, molecular and sequencing-based techniques have emerged as complementary tools for the diagnosis of invasive fungal infections. PCR-based assays, including pan-fungal and genus-specific approaches (e.g., Aspergillus PCR) have demonstrated variable performance depending on the clinical context, fungal species, and methodological standardization. Metagenomic next-generation sequencing (mNGS) has also gained increasing attention as a promising diagnostic modality, enabling the unbiased detection of multiple pathogens from a single clinical sample (e.g., blood or bronchoalveolar lavage). This approach may be particularly useful in cases of atypical presentations, rare fungal pathogens, or mixed infections. However, the clinical interpretation of mNGS results remains challenging, as standardized thresholds for defining infection based on sequencing reads have not yet been established. Although these assays may improve diagnostic sensitivity, their availability and routine implementation remain limited in many centres, particularly in low- and middle-income settings [81,119].

Finally, positron emission tomography combined with computed tomography (PET-CT) can be useful as a complementary tool in selected cases, particularly for assessing treatment response and guiding the duration of therapy in deep-seated or disseminated fungal infections. Its ability to detect metabolically active lesions is valuable for differentiating active infection from residual structural abnormalities [113,120].

## 7. Step-Down Strategies and Future Directions

The role of combination antifungal therapy in breakthrough infections remains complex and, in many scenarios, controversial. While combination regimens, such as azole plus echinocandin or polyene-based combinations, have been explored in specific settings (e.g., invasive aspergillosis or mucormycosis), robust evidence supporting their routine use is limited and largely derived from selected populations or observational studies. In this review, combination therapy is primarily discussed as a pragmatic, short-term strategy to broaden antifungal coverage in the initial management of suspected breakthrough infections, particularly in profoundly immunocompromised patients at risk for infections caused by resistant yeasts, molds, or mixed fungal pathogens [121,122].

**Figure 2:**
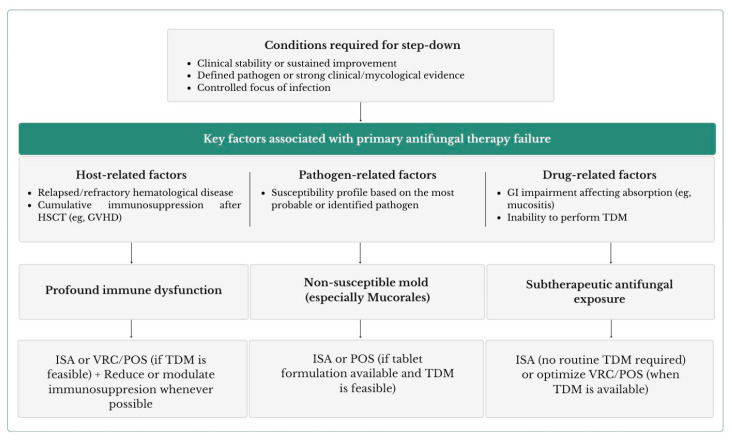
Step-down strategies from L-Amb to triazoles after initial clinical response in breakthrough mold infections. This figure applies to proven or highly probable breakthrough mold infections, after initial empirical management and diagnostic evaluation, and integrates host, pathogen, and pharmacological determinants to guide targeted therapy and step-down decisions. Abbreviations: HSCT = Hematopoietic stem cell transplant, GVHD = Graft-versus-host disease, TDM = Therapeutic drug monitoring, GI = Gastrointestinal, ISA = Isavuconazole, VRC = Voriconazole, POS = Posaconazole.

Once clinical stability is achieved, identifying the key determinants of primary therapy failure, integrating host, pathogen, and pharmacological factors, is essential to guide step-down decisions, as illustrated in Figure 2 [25,121]. Reduction of immunosuppression when feasible, optimisation of antifungal exposure guided by TDM, and management of concomitant infections are critical components of care [19,104]. In parallel, there is growing interest in immunomodulatory strategies aimed at restoring or enhancing antifungal host responses as adjuncts to antifungal therapy [122,123].

Targeted oral therapy depends on the identified pathogen and susceptibility profile. Voriconazole, isavuconazole, and posaconazole remain first-line options for IA, whereas isavuconazole and posaconazole are approved step-down therapies for mucormycosis following L-AmB induction [124,125]. Isavuconazole has consistently demonstrated lower hepatotoxicity compared with other triazoles in RCTs and real-world cohorts, an important advantage in patients receiving hepatotoxic chemotherapy or immunosuppressants [8,124,126]. After step-down, continued radiological reassessment, monitoring of antifungal exposure, and evaluation of immune recovery are required to guide treatment duration and decisions regarding secondary prophylaxis [109,127,128,129].

However, limited step-down options are available for treating intrinsically resistant molds such as *Lomentospora* spp. [89,109]. This limited antifungal armamentarium against resistant fungi reinforces the urgent need for novel agents. Several compounds in advanced development, including ibrexafungerp, rezafungin, olorofim, and fosmanogepix, are in advanced development and hold potential to significantly expand IFI management and to expand future management options, particularly in the setting of antifungal resistance and pharmacological limitations (Supplementary Table S1) [127,128,129,130,131].

## 8. General Management Principles and Conclusions

The emergence of antifungal resistance, together with pharmacological and diagnostic limitations, poses additional challenges in bIFIs, remaining a significant cause of morbidity and mortality among hematologic patients and HSCT recipients [25,97,132]. Effective management requires a coordinated, stepwise strategy that prioritizes early recognition, prompt empirical escalation to broad-spectrum antifungal therapy (most commonly L-AmB-based regimens, alone or in combination with triazoles), and an aggressive diagnostic workup (including imaging, biomarkers, BAL, and biopsy when feasible). Rapid transition to targeted therapy guided by species identification, antifungal susceptibility testing, and therapeutic drug monitoring is essential to optimize outcomes (Supplementary Tables S2 and S3) [19,83,121].

An intensive diagnostic evaluation is crucial not only to guide optimal acute management but also to guide secondary antifungal prophylaxis during subsequent phases of immunosuppression [133,134,135]. In patients receiving anti-mold azole prophylaxis, L-AmB remains the preferred initial empirical option, with step-down to an oral azole once clinical stability is achieved and susceptibility data are available [19,25,108,134]. In centres without routine TDM, isavuconazole represents an attractive alternative due to its predictable pharmacokinetics and favourable safety profile [104,108].

Future research is needed to focus on refining epidemiological surveillance of bIFIs across regions and clinical settings, expanding access to high-quality mycology diagnostics and susceptibility testing, clarifying the role of emerging antifungals, and defining optimal treatment duration and prevention strategies to improve long-term outcomes in this vulnerable population.

## 9. Key Messages

- Breakthrough invasive fungal infection is a frequent and severe complication among high-risk hematologic patients;
- Main drivers of bIFI are profound immunosuppression, subtherapeutic antifungal exposure, selective pressure favouring intrinsically resistant fungi, and emergence of acquired resistance;
- Management priorities includes rapid and accurate diagnosis, antifungal susceptibility testing, and prompt initiation of effective therapy;
- Liposomal amphotericin B is the preferred first-line agent following azole prophylaxis; step-down to an oral azole can be considered once patients become clinically stable, with isavuconazole favoured where therapeutic drug monitoring is unavailable;
- Further research is needed to refine epidemiology, improve diagnostic and laboratory tools, evaluate novel antifungals, and define optimal treatment duration to enhance patient outcomes.

## 10. Limitations

As a narrative review, this work is subject to potential selection bias in the literature included and reflects, in part, the expert opinion of the author group. Additionally, although we aimed to incorporate both global and Latin American data, the epidemiological patterns described may not be fully representative of all centres within the region, particularly given the heterogeneity in diagnostic capacity and access to antifungal therapies.

## Data Availability

The original contributions presented in this study are included in the article/Supplementary Material. Further inquiries can be directed to the corresponding author.

## Supplementary Materials

The following supporting information can be downloaded at: https://www.mdpi.com/article/10.3390/microorganisms14040904/s1, Supplementary Table S1: New antifungal agents mechanisms, spectrum, and development status; Supplementary Table S2: General antifungal susceptibility patterns of key fungal pathogens; Supplementary Table S3: Therapeutic options and considerations for specific fungal pathogens [20,64,66,70,89,109,128,131,132,136,137,138,139,140,141,142,143,144,145,146,147,148,149,150,151,152].

## Abbreviations

The following abbreviations are used in this manuscript:

IFI	Invasive fungal infection
HSCT	Hematopoietic stem cell transplantation
IC	Invasive candidemia
IA	Invasive aspergillosis
bIFI	Breakthrough invasive fungal infection
GVHD	Graft-versus-host disease
CMV	Cytomegalovirus
DDI	Drug–drug interaction
GM	Galactomannan
CT	Computed tomography
RCT	Randomized clinical trials
AML	Acute myeloid leukemia
MDS	Myelodysplastic syndrome
EORTC/MSG-ERC	European Organization for Research and Treatment of Cancer and the Mycoses Study Group Education and Research Consortium
GI	Gastrointestinal
MDR	Multidrug-resistant
AST	Antifungal susceptibility testing
TDM	Therapeutic drug monitoring
L-AmB	Liposomal amphotericin B
BAL	Bronchoalveolar lavage
mNGS	Metagenomic next-generation sequencing
