# Supplementary material for: Managing Breakthrough Fungal Infections in Hematologic Patients: Determinants and Practical Management from a Latin American Perspective on Behalf of INFOCUS LATAM–ISHAM Working Group
Source: Microorganisms. 2026 Apr 16;14(4):904. doi: 10.3390/microorganisms14040904 (PMC13118672; doi:10.3390/microorganisms14040904)
Supplement: Supplementary file 1 [file microorganisms-14-00904-s001.zip › microorganisms-4202685-supplementary.pdf]

Supplementary Materials

Managing Breakthrough Fungal Infections in Haematologic Patients: Determinants and Practical Management from a Latin American Perspective on behalf of INFOCUS LATAM–ISHAM Working Group

Supplementary Table S1. New antifungal agents mechanisms, spectrum, and development status.

Agent	Mechanism of Action	Antifungal Spectrum	Formulation	Stage of Clinical Development/Current Status
Ibrexafungerp [1–3]	First-in-class triterpenoid inhibitor of β-(1,3)-D-glucan synthase, structurally distinct from echinocandins, disrupting fungal cell wall synthesis.	Broad activity against *Candida spp*., including echinocandin-resistant, FKS-mutant and *Candida auris* strains; limited activity against moulds	Oral and intravenous formulations	FDA-approved for *Candida* spp. vulvovaginitis; Phase III for invasive candidiasis and difficult-to-treat fungal infections.
Rezafungin [3,4]	Next-generation echinocandin inhibitor of β-(1,3)-D-glucan synthase with prolonged half-life, preventing the production of an essential fungal cell wall component; designed for a long half-life.	*Candida* spp. (including many resistant isolates) and *Aspergillus* spp.; limited activity against Mucorales and rare moulds.	Intravenous, once-weekly dosing	FDA and EMA approved for Candidemia and Invasive Candidiasis; Phase III clinical trials ongoing for antifungal prophylaxis in HSCT recipients.
Olorofim [3,5]	First-in-class orotomide; selectively inhibits the fungal dihydroorotate dehydrogenase (DHODH) enzyme, disrupting pyrimidine biosynthesis.	Active against *Aspergillus* spp. (including some azole-resistant isolates), *Scedosporium* spp., and *Lomentospora prolificans*; no relevant activity against *Candida* spp. or Mucorales.	Oral	Phase III for invasive aspergillosis; Phase IIb for rare or refractory mould infections; granted FDA breakthrough therapy designation.
Fosmanogepix [3,6,7]	First-in-class inhibitor of Gwt1, disrupting glycosylphosphatidylinositol (GPI)-anchor biosynthesis and fungal cell wall/membrane integrity.	Broad activity against *Candida spp., Aspergillus spp., Fusarium spp., Scedosporium spp., and Lomentospora spp.*; activity against some multidrug-resistant pathogens, including *Candida auris.*	Oral and intravenous formulations	Phase II completed for Candidemia, *C. auris*, and rare molds; Phase III clinical trials planned or ongoing for Invasive Candidiasis.

Supplementary Table S2. General antifungal susceptibility patterns of key fungal pathogens.

Fungal Pathogen	Amphotericin B	Voriconazole	Posaconazole	Isavuconazole	Echinocandins	Primary Resistance Mechanism	Source
Candida glabrata/ Nakaseomyces glabratus	++	+/-	+/-	+/-	++	FKS1/2 mutations; Efflux pumps (CDR1/2).	[8,9]
Candida krusei/ Pichia kudriavzevii	++	+	+	+	++	CYP51A alterations; FKS1 mutations.	[8,9]
Candida auris/ Candidozyma auris	+/-	+/-	+/-	+/-	+	FKS1/2 & ERG11 (Y132F) mutations; CDR1 efflux.	[8,9]
Aspergillus fumigatus	++	++	++	++	+/- (F)	CYP51A mutations (TR34/L98H, TR46).	[10–12]
Other *Aspergillus spp.*	+/-	++	++	++	+/- (F)	CYP51A mutations; Intrinsic R in *A. terreus.*	[13,14]
Mucorales	++	0	++	++	0	Lack of target affinity (CYP51, β-glucan).	[15–17]
Fusarium spp.	+	+	+	+	0	Reduced target affinity; Intrinsic R to echinocandins.	[18]
Scedosporium apiospermum	0	+	+	+	0	Intrinsic R to polyenes and echinocandins.	[19]
Lomentospora prolificans	0	+/-	+/-	+/-	0	Pan-antifungal resistance; efflux systems.	[18]
Trichosporon spp.	+/-	++	++	++	0	Lack of 1,3-β-D-glucan synthase target.	[20,21]
Rhodotorula spp.	++	+/-	+/-	+/-	0	Lack of β-glucan synthase activity.	[22,23]
Geotrichum spp.	+/-	+/-	+/-	+/-	0	Cell wall composition; lack of β-glucan synthase.	[22,23]

Legend:

++	Usually more susceptible (reliable *activity in vitro*)/ Preferred
+	Less susceptible (variable activity *in vitro) or more adverse events (AMB)*/ Alternative
+/-	Variable susceptibility; (F) fungistatic/ Limited utility
0	Intrinsically resistance / Avoid use

Obs: These categorizations are intended to provide a simplified, clinically oriented overview based on available in vitro data and guideline recommendations.

Supplementary Table S3. Therapeutic options and considerations for specific fungal pathogens.

Fungal Pathogen	First-Line Therapy	Alternative Therapy	Comment	Source
Candida spp.	Echinocandins	L-AmB	Knowledge on local epidemiology should be considered when choosing second-line treatment (for either *C. glabrata/N. glabratus, C. auris or Candida krusei/ Pichia kudriavzevi.*	[9]
Aspergillus spp.	VRC or ISA	L-AmB; Voriconazole + echinocandin combination; PCZ	*A. terreus* or *A. alliaceus* (*A. flavus* complex): Avoid L-AmB<br>*A. lentulus* (*A. fumigatus* complex): Avoid azole monotherapy<br>*A. niger* complex: Consider VRC instead of ISA (MIC dilution generally higher)	[14,24–26]
Mucorales	L-AmB	ISA, PCZ	ISA therapy associated with similar outcomes compared to L-AmB; PCZ mainly used as secondary or salvage therapy; Early and aggressive surgical debridement is critical.	[17,27]
Fusarium spp.	L-AmB or VRC (alone or in combination)	L-AmB/VRC in combination with TBF; PCZ; ISA	Variable in vitro activity for triazoles without correlation with clinical outcomes. VRC and L-AmB equally effective in retrospective studies.	[18,27]
Scedosporium apiospermum	VRC	PCZ or ISA according to AST results	VRC most active in vitro; Variable in vitro activity of other triazoles (PCZ, ITZ,ISA). Avoid L-AmB.	[18,27]

Lomentospora prolificans	VRC+TBF	VRC or TBF with another ISA; POS or ITR	Extremely multidrug-resistant (pan-resistant) to nearly all available antifungals; VRC + TBF is associated with better outcomes; Avoid L-AmB. Olorofim is a first-in-class agent showing promising results in salvage settings. Fosmanogepix also demonstrates potent activity.	[18,27]
Trichosporon spp.	VRC	PCZ	L-AmB (Marginal recommendation); Avoid Echinocandins.	[22]
Rhodotorula spp.	L-AmB	VRC	L-AmB with or without flucytosine (moderately recommended); Avoid echinocandins and triazoles.	[22]
Geotrichum spp.	L-AmB	VRC	L-AmB with or without flucytosine (moderately recommended); Avoid echinocandins.	[22]

Abbreviations: Liposomal Amphotericin B (L-AmB); Voriconazole (VRC); Posaconazole (PCZ); Itraconazole (ITR); Isavuconazole (ISA); Terbinafine (TBF).

References

1. Angulo, D.A.; Alexander, B.; Rautemaa-Richardson, R.; Alastruey-Izquierdo, A.; Hoenigl, M.; Ibrahim, A.S.; Ghannoum, M.A.; King, T.R.; Azie, N.E.; Walsh, T.J. Ibrexafungerp, a Novel Triterpenoid Antifungal in Development for the Treatment of Mold Infections. *J. Fungi* **2022**, *8*, 1121.
2. El Ayoubi, L.W.; Allaw, F.; Moussa, E.; Kanj, S.S. Ibrexafungerp: A narrative overview. *Curr. Res. Microb. Sci.* **2024**, *6*, 100245.
3. Hoenigl, M.; Sprute, R.; Egger, M.; Arastehfar, A.; Cornely, O.A.; Krause, R.; Lass-Flörl, C.; Prattes, J.; Spec, A.; Thompson GR 3rd; et al. The Antifungal Pipeline: Fosmanogepix, Ibrexafungerp, Olorofim, Opelconazole, and Rezafungin. *Drugs* **2021**, *81*, 1703–1729.
4. Thompson, G.R.; 3rd; Soriano, A.; Honore, P.M.; Bassetti, M.; Cornely, O.A.; Kollef, M.; Kullberg, B.J.; Pullman, J.; Hites, M.; Fortún, J.; et al. Efficacy and safety of rezafungin and caspofungin in candidaemia and invasive candidiasis: Pooled data from two prospective randomised controlled trials. *Lancet Infect. Dis.* **2024**, *24*, 319–328.
5. Maertens, J.A.; Thompson, G.R.; 3rd; Spec, A.; Donovan, F.M.; Hammond, S.P.; Bruns, A.H.W.; Rahav, G.; Shoham, S.; Johnson, R.; Rijnders, B.; et al. Olorofim for the treatment of invasive fungal diseases in patients with few or no therapeutic options: A single-arm, open-label, phase 2b study. *Lancet Infect. Dis.* **2025**, *25*, 1177–1188.
6. Alkhazraji, S.; Gebremariam, T.; Alqarihi, A.; Gu, Y.; Mamouei, Z.; Singh, S.; Wiederhold, N.P.; Shaw, K.J.; Ibrahim, A.S. Fosmanogepix (APX001) Is Effective in the Treatment of Immunocompromised Mice Infected with Invasive Pulmonary Scedosporiosis or Disseminated Fusariosis. *Antimicrob. Agents Chemother.* **2020**, *64*, e01735-19.
7. Lamoth, F.; Lewis, R.E.; Kontoyiannis, D.P. Investigational Antifungal Agents for Invasive Mycoses: A Clinical Perspective. *Clin. Infect. Dis.* **2022**, *75*, 534–544. Erratum in *Clin. Infect. Dis.* **2023**, *76*, 779.
8. Colombo, A.L.; Júnior, J.N.; Guinea, J. Emerging multidrug-resistant Candida species. *Curr. Opin. Infect. Dis.* **2017**, *30*, 528–538.
9. Cornely, O.A.; Sprute, R.; Bassetti, M.; Chen, S.C.; Groll, A.H.; Kurzai, O.; Lass-Flörl, C.; Ostrosky-Zeichner, L.; Rautemaa-Richardson, R.; Revathi, G.; et al. Global guideline for the diagnosis and management of candidiasis: An initiative of the ECMM in cooperation with ISHAM and ASM. *Lancet Infect. Dis.* **2025**, *25*, e280–e293.
10. Beardsley, J.; Halliday, C.L.; Chen, S.C.; Sorrell, T.C. Responding to the emergence of antifungal drug resistance: Perspectives from the bench and the bedside. *Future Microbiol.* **2018**, *13*, 1175–1191.
11. Escribano, P.; Recio, S.; Peláez, T.; Bouza, E.; Guinea, J. *Aspergillus fumigatus* strains with mutations in the cyp51A gene do not always show phenotypic resistance to itraconazole, voriconazole, or posaconazole. *Antimicrob. Agents Chemother.* **2011**, *55*, 2460–2462.
12. Morrissey, C.O.; Kim, H.Y.; Duong, T.N.; Moran, E.; Alastruey-Izquierdo, A.; Denning, D.W.; Perfect, J.R.; Nucci, M.; Chakrabarti, A.; et al. Aspergillus fumigatus-a systematic review to inform the World Health Organization priority list of fungal pathogens. *Med. Mycol.* **2024**, *62*, myad129.

13. Pontes, L.; Gualtieri Beraquet, C.A.; Arai, T.; Watanabe, A.; Moretti, M.L.; Schreiber, A.Z. Selection of *Aspergillus fumigatus* isolates carrying the G448S substitution in CYP51A gene after long-term treatment with voriconazole in an immunocompromised patient. *Med. Mycol. Case Rep.* **2022**, *36*, 5–9.
14. Ullmann, A.J.; Aguado, J.M.; Arikan-Akdagli, S.; Denning, D.W.; Groll, A.H.; Lagrou, K.; Lass-Flörl, C.; Lewis, R.E.; Munoz, P.; Verweij, P.E.; et al. Diagnosis and management of Aspergillus diseases: Executive summary of the 2017 ESCMID-ECMM-ERS guideline. *Clin. Microbiol. Infect.* **2018**, *24*, e1–e38.
15. Axell-House, D.B.; Wurster, S.; Jiang, Y.; Kyvernitakis, A.; Lewis, R.E.; Tarrand, J.J.; Raad, I.I.; Kontoyiannis, D.P. Breakthrough Mucormycosis Developing on Mucorales-Active Antifungals Portrays a Poor Prognosis in Patients with Hematologic Cancer. *J. Fungi* **2021**, *7*, 217.
16. Morrissey, C.O.; Kim, H.Y.; Garnham, K.; Dao, A.; Chakrabarti, A.; Perfect, J.R.; Alastruey-Izquierdo, A.; Harrison, T.S.; Bongomin, F.; Galas, M.; et al. Mucorales: A systematic review to inform the World Health Organization priority list of fungal pathogens. *Med. Mycol.* **2024**, *62*, myad130.
17. Cornely, O.A.; Alastruey-Izquierdo, A.; Arenz, D.; Chen, S.C.A.; Dannaoui, E.; Hochhegger, B.; Hoenigl, M.; Jensen, H.E.; Lagrou, K.; Lewis, R.E.; et al. Global guideline for the diagnosis and management of mucormycosis: An initiative of the European Confederation of Medical Mycology in cooperation with the Mycoses Study Group Education and Research Consortium. *Lancet Infect. Dis.* **2019**, *19*, e405–e421.
18. Hoenigl, M.; Salmanton-García, J.; Walsh, T.J.; Nucci, M.; Neoh, C.F.; Jenks, J.D.; Lackner, M.; Sprute, R.; Al-Hatmi, A.M.S.; Bassetti, M.; et al. Global guideline for the diagnosis and management of rare mould infections: An initiative of the European Confederation of Medical Mycology in cooperation with the International Society for Human and Animal Mycology and the American Society for Microbiology. *Lancet Infect. Dis.* **2021**, *21*, e246–e257. Erratum in *Lancet Infect. Dis.* **2021**, *21*, e81.
19. Hoenigl, M.; Salmanton-García, J.; Walsh, T.J.; Nucci, M.; Neoh, C.F.; Jenks, J.D.; Lackner, M.; Sprute, R.; Al-Hatmi, A.M.S.; Bassetti, M.; et al. Global guideline for the diagnosis and management of rare mould infections: An initiative of the European Confederation of Medical Mycology in cooperation with the International Society for Human and Animal Mycology and the American Society for Microbiology. *Lancet Infect. Dis.* **2021**, *21*, e246–e257. Erratum in *Lancet Infect. Dis.* **2021**, *21*, e81.
20. Almeida, J.N.d.; Jimenez-Ortigosa, C.; Francisco, E.C.; Colombo, A.L.; Perlin, D.S. *ERG11* Analysis among Clinical Isolates of Trichosporon asahii with Different Azole Susceptibility Profiles. *Antimicrob. Agents Chemother.* **2022**, *66*, e01101-22.
21. Colombo, A.L.; Padovan, A.C.; Chaves, G.M. Current knowledge of *Trichosporon* spp. and Trichosporonosis. *Clin. Microbiol. Rev.* **2011**, *24*, 682–700.
22. Chen, S.C.; Perfect, J.; Colombo, A.L.; Cornely, O.A.; Groll, A.H.; Seidel, D.; Albus, K.; de Almedia, J.N., Jr.; Garcia-Effron, G.; Gilroy, N.; et al. Global guideline for the diagnosis and management of rare yeast infections: An initiative of the ECMM in cooperation with ISHAM and ASM. *Lancet Infect. Dis.* **2021**, *21*, e375–e386. Erratum in *Lancet Infect. Dis.* **2024**, *24*, e485.
23. Sprute, R.; Cornely, O.A.; Chen, S.C.; Seidel, D.; Schuetz, A.N.; Zhang, S.X. All You Need To Know and More about the Diagnosis and Management of Rare Yeast Infections. *mBio* **2021**, *12*, e0159421.
24. Jenks, J.D.; Hoenigl, M. Treatment of Aspergillosis. *J. Fungi* **2018**, *4*, 98.
25. Tissot, F.; Agrawal, S.; Pagano, L.; Petrikkos, G.; Groll, A.H.; Skiada, A.; Lass-Flörl, C.; Calandra, T.; Viscoli, C.; Herbrecht, R. ECIL-6 guidelines for the treatment of invasive candidiasis, aspergillosis and mucormycosis in leukemia and hematopoietic stem cell transplant patients. *Haematologica* **2017**, *102*, 433–444.
26. Dadwal, S.S.; Hohl, T.M.; Fisher, C.E.; Boeckh, M.; Papanicolaou, G.; Carpenter, P.A.; Fisher, B.T.; Slavin, M.A.; Kontoyiannis, D.P. American Society of Transplantation and Cellular Therapy Series, 2: Management and Prevention of Aspergillosis in Hematopoietic Cell Transplantation Recipients. *Transpl. Cell. Ther.* **2021**, *27*, 201–211.
27. Douglas, A.P.; Lamoth, F.; John, T.M.; Groll, A.H.; Shigle, T.L.; Papanicolaou, G.A.; Chemaly, R.F.; Carpenter, P.A.; Dadwal, S.S.; Walsh, T.J.; Kontoyiannis, D.P. American Society of Transplantation and Cellular Therapy Series: #8-Management and Prevention of Non-Aspergillus Molds in Hematopoietic Cell Transplantation Recipients. *Transpl. Cell. Ther.* **2025**, *31*, 194–223.

Disclaimer/Publisher's Note: The statements, opinions and data contained in all publications are solely those of the individual author(s) and contributor(s) and not of MDPI and/or the editor(s). MDPI and/or the editor(s) disclaim responsibility for any injury to people or property resulting from any ideas, methods, instructions or products referred to in the content.
